# Psychometric evidence of the WHO-5 well-being index in a sample of participants from hospitals and older adults care centers in Peru

**DOI:** 10.3389/fpubh.2025.1670429

**Published:** 2025-11-13

**Authors:** Patricia del Pilar Díaz Gamarra, Fernando Joel Rosario Quiroz, Erika Roxana Estrada Alomía, Noemí Edith Iparraguirre Yaurivilca, Miguel Angel Misare Condori

**Affiliations:** Universidad Cesar Vallejo, Trujillo, Peru

**Keywords:** general well-being, family functioning, older adults, psychometric properties, reliability

## Abstract

**Introduction:**

The study of general well-being in older adulthood is of vital importance due to the negative repercussions of aging on daily life. Therefore, the objective of this research was to analyze the psychometric properties of the WHO-5 well-being index in a sample of participants from hospitals and older care centers in Peru.

**Method:**

A total of 661 older adults from Metropolitan Lima participated in the study (65% women and 35% men), aged between 60 and 93 years. They concurrently completed the WHO-5 well-being index and the Family APGAR.

**Results:**

The findings demonstrated adequate fit indices for the original five-item model: *χ*^2^/df = 3.73, CFI = 0.998, TLI = 0.995, SRMR = 0.03, and RMSEA = 0.06. Factor loadings were above 0.50. Convergent validity, assessed through Pearson’s correlation coefficient, was significant and direct (*r* = 0.35). Internal consistency indices (*α* = 0.84; *ω* = 0.84) were satisfactory. The unidimensional structure of the instrument was confirmed, as well as its measurement invariance across sexes.

**Discussion and conclusions:**

The WHO-5 well-being index, when applied to older adults in Peru, confirms its unidimensional structure, demonstrates evidence of validity and reliability, and is an equivalent measure across sexes. This suggests its utility as a brief, reliable instrument for evaluating well-being in this population.

## Introduction

### Study context

Research on subjective well-being in older adults has shown that social and emotional factors play a central role in healthy aging. In Europe, loneliness and social isolation are associated with sustained declines in well-being (1, 108, 112, 114), whereas community-based programs and positive psychology interventions exhibit significant protective effects (2). In Latin America, studies in Chile highlight the close relationship between self-perceived health, functional capacity, and social support with higher levels of well-being (3, 113), and in Mexico, non-contributory pensions have been documented to produce significant improvements in the perception of well-being in old age (4). Similarly, comparisons between Costa Rica and Spain have underscored the influence of cultural factors in shaping successful aging (5). In the Peruvian case, the recent adaptation of the WHO-5 into Quechua represents an important intercultural advancement (6); however, psychometric studies supporting its use among Spanish-speaking older adults are still lacking, revealing an empirical gap and providing justification for the present study.

Recent research confirms that the quality of life of older adults depends on institutional and community factors (110), with significant differences observed across European countries such as Poland, Germany, and Austria (7). In Lithuania, the need for specific policies to improve quality of life has been documented (8), while in Spain, differences in residence area (urban vs. rural) generate variations in health, lifestyle, and well-being (9). Internationally, social and community support are determinants of subjective well-being (10, 109). Community dining programs and participation in social activities reinforce belongingness and life satisfaction (11). Psychological resilience, promoted through body–mind exercise programs, has also been identified as a key protective mechanism (12, 116).

### Concepts and indicators of well-being

The concept of well-being has evolved from a definition centered on the absence of disease to a holistic approach integrating biological, social, mental, environmental, and economic aspects (13). Traditionally, objective indicators such as income level, life expectancy, and literacy have been used (14). However, subjective measures have also been employed, such as perceived quality of life, levels of anxiety and depression, life satisfaction, and happiness (15–20, 107).

Recent studies emphasize the importance of contextual and material factors for older adults’ well-being. In Greece, physical activity has been associated with better quality of life (21), while in the European Union, living conditions significantly affect well-being (22). Public health research shows that subjective well-being can be understood as a multidimensional construct influenced by health literacy and territorial context (23). At the individual level, anxiety and depression predict negative trajectories of well-being (24), and loneliness exacerbates the effects of ageism in widowed populations (25). Conversely, older adults demonstrate resilience and emotional stability in the face of adversity (26), and positive attitudes toward aging are linked to greater overall well-being (27).

### WHO-5 as a measure of well-being

The WHO-5 well-being index, developed by the WHO, consists of five items that assess subjective well-being globally and simply. It is recommended as a depression screening tool in primary care (28–30).

The content of the WHO-5 is directly linked to the hedonic-oriented model of subjective well-being, as it focuses on the frequency of positive affective experiences. The first item, referring to feeling cheerful and in good spirits, reflects the central component of positive affect described by Diener (31) and Watson, Clark, and Tellegen (32). The second item, concerning feeling calm and relaxed, assesses the experience of emotional tranquility and the absence of distress, in line with the concept of positive health proposed by the WHO (33) and the clinical interpretation of Bech et al. (34). The third item, related to energy and vitality, connects with self-determination theory, which considers vitality a central indicator of well-being (35). The fourth item, associated with waking up feeling rested, integrates a somatic component of subjective well-being linked to healthy functioning and physiological recovery (36). Finally, the fifth item, which explores interest in daily life, alludes to motivation and life purpose, dimensions that combine the hedonic perspective with life satisfaction as described in broader models of well-being (37, 117). Taken together, these items enable a stepped assessment that ranges from basic emotional experience to life engagement, providing evidence of coherence with contemporary models of psychological well-being.

Multiple studies have confirmed its psychometric validity across countries and contexts, showing internal consistency and good fit to the unidimensional model (19, 29, 38–48, 111). Likewise, it has proven useful in detecting depression in various medical conditions (28, 49–58) and in different age groups (30, 59–65).

### Research gap and study objective

The WHO-5, brief and easy to administer, has demonstrated validity among Spanish-speaking older adults and has been adapted into Quechua in Peru, enabling its use in both urban and rural contexts. Its unidimensional structure and regional invariance support comparisons across socioeconomic levels, and its utility as a screening tool for depression underpins its application in both community and clinical settings (6, 65–68). However, despite this international and regional evidence, no studies have specifically examined the psychometric properties of the WHO-5 among Spanish-speaking older adults in Peru. This gap limits the availability of a brief, reliable, and culturally relevant instrument to assess well-being in a highly vulnerable group. Consequently, the present study aims to analyze the psychometric evidence of the WHO-5 well-being index in a Peruvian sample of Spanish-speaking older adults, with the purpose of contributing to early detection and supporting strategies for mental health care and well-being.

## Method

### Participants

The sample consisted of 661 older adults, with 64.9% women and 35.1% men, aged between 60 and 93 years (*M* = 70.09; SD = 7.33). Regarding marital status, 46.4% were married, 10.6% cohabiting, 22.1% widowed, 13.4% separated or divorced, and 7.4% single. Household composition was as follows: 12.1% lived alone, 12.9% with a partner, 26.2% with children, 33% with partner and children, and 15.9% with other relatives. Educational levels were diverse: 7.6% did not complete primary school, 13.2% completed primary school, 3% incomplete secondary, 26.5% completed secondary, 2.3% incomplete technical studies, 11.5% completed technical studies, 12% incomplete university studies, 20.3% completed university studies, and 3.8% had postgraduate education. Additionally, 51.7% attended Older Adults Care Centers (CAM), while 48.3% did not.

Inclusion criteria were: being aged 60 years or older, residing in the community or attending older adults care centers, having the capacity to autonomously respond to questionnaires, and providing written or digital informed consent. Exclusion criteria were severe cognitive impairment or confirmed dementia diagnosis, as well as acute medical conditions that limited participation, ensuring stable health at the time of evaluation.

Recruitment was conducted through direct invitations at hospitals, older adults care centers (CAM), and community spaces, complemented by virtual dissemination through institutional support networks.

A non-probabilistic convenience sampling method was used, ensuring voluntariness and anonymity after informed consent. Only participants who fully completed the evaluation protocol were included in the analysis. The sample size (n = 661) was deemed adequate for robust psychometric analysis, considering that categorical data precision improves with ≥300 cases, particularly with ordinal scales, classified as ‘very good’ according to Bandalos (69). Although there is no consensus on the ideal sample size for psychometric studies, Arafat et al. (70) recommend at least 500 participants for validation, while Newsom (71) suggests >400 when using MLR estimation and >500 for DWLS or WLSMV estimators. Therefore, the sample size in this study was appropriate.

### Instruments

*Sociodemographic questionnaire*: collected information on age, sex, marital status, household composition, educational level, and participation in older adults permission to use the instrument was obtained care centers. These indicators contextualized well-being factors, given the influence of sociodemographic variables on quality of life and mental health in older adults (27, 72, 73).

*WHO-5 well-being index*: a self-report instrument developed by the WHO (33) to assess depressive symptoms through five items related to energy, interest, and mood, using a 4-point ordinal scale (0 = never, 1 = sometimes, 2 = often, 3 = always) (74). This study used the Spanish version validated by Simancas-Pallares et al. (75), which reported internal reliability of *ω* = 0.877 and *α* = 0.852, with a factorial structure explaining 56.17% of total variance, demonstrating adequate functioning.

*Family APGAR*: developed by Smilkstein in 1978, this instrument assesses family functioning through five items related to participation, resource gradient, adaptation, affection, and problem-solving capacity, with a 5-point Likert-type scale (0 = never, 1 = hardly ever, 2 = sometimes, 3 = almost always, 4 = always) (76).

### Procedure

The study followed an instrumental design aimed at analyzing the psychometric properties of the WHO-5 in older adults. Permission to use the instrument was obtained from the authors. Data were collected using two modalities: (a) a face-to-face protocol including informed consent, sociodemographic questionnaire, and instruments, and (b) a virtual form. In the online modality, participants were presented with the study’s objective, the voluntary and anonymous nature of participation, and the informed consent form, with only those providing affirmative consent proceeding to the evaluation protocol.

Data collection spanned 6 months. Protocols not meeting inclusion criteria were excluded. Data were systematized in Excel and exported to Jamovi and RStudio for analysis. The study was funded by the Faculty Research Fund of Universidad César Vallejo (Resolution RVI No. P-2024-017 -VI-UCV, July 31, 2024) and approved by the university’s ethics committee (PID No. 003–2024, April 10, 2024).

### Data analysis

The study conducts a psychometric analysis, which is essential to ensure that an instrument measures the intended construct with validity and reliability. This process allows for the verification of internal structure, consistency, and comparability by sex, thereby preventing interpretative bias. In specific populations such as older adults, this type of evidence is critical to guarantee clinical relevance and practical utility (77).

Statistical analyses were conducted with RStudio (version 4.1.2) and Jamovi (version 2.3.2.6). A polychoric correlation matrix was calculated, suitable for ordinal items, following recommendations by Gadermann et al. (78), Jöreskog (79), and Viladrich et al. (80). Item analysis criteria included corrected item-total correlations (IHC) > 0.30 (81), and skewness and kurtosis values between ±1.50 (82). Significant correlations with an external criterion were also examined.

Internal structure validity was evaluated through confirmatory factor analysis (CFA) using the robust WLSMV estimator, recommended for ordinal data. Model fit indices were assessed with RMSEA ≤ 0.08, CFI ≥ 0.95, TLI ≥ 0.90, and χ^2^/df, following Escobedo et al. (83). Convergent validity required significant correlations (*p* < 0.01) with external variables. Factorial invariance was examined using ΔCFI > 0.010 and ΔRMSEA < 0.015 cutoffs (84). Reliability was assessed with McDonald’s omega (85), with acceptable values between 0.70 and 0.90 (86), though values above 0.65 may be accepted in specific cases (87).

## Results

This section presents the psychometric analysis results of the WHO-5 well-being index applied to Peruvian older adults. Findings include polychoric correlations, item analysis, confirmatory factor analysis, factorial invariance by sex, validity with family functioning, reliability indices, and reference percentiles. These outcomes demonstrate the instrument’s internal consistency, validity, and applicability within the studied population.

### Polychoric correlation matrix

Table 1 presents the polychoric correlations obtained among the five items of the WHO-5 well-being index. Polychoric correlations are appropriate for evaluating the relationship between ordinal categorical variables, as is the case with Likert-scale items. The correlations ranged from 0.44 (W1 and W5) to 0.70 (W1 and W2), indicating moderate to high levels of association among the items. These values show that there is no problem of collinearity, meaning that redundancy between items is not present (88). This pattern suggests that the items are interrelated, reflecting a possible unidimensional structure of the evaluated construct. These findings support the internal consistency of the instrument and suggest that the items measure related aspects of the same construct.

**Table 1:** Polychoric correlations among items of the WHO-5 well-being index.

Items	W1	W2	W3	W4	W5
W1	1				
W2	0.70	1			
W3	0.64	0.66	1		
W4	0.63	0.65	0.69	1	
W5	0.44	0.45	0.58	0.55	1

### Item analysis of the WHO-5 in Peruvian older adults

Table 2 presents a detailed analysis of the five items of the WHO-5 well-being index, evaluating their psychometric characteristics. Response percentages for each Likert-scale category are reported, along with descriptive measures such as mean, standard deviation (SD), skewness, and kurtosis. None of the response percentages exceeded 80%, indicating the absence of bias. Regarding response frequencies, all were above 10, which is optimal since frequencies below this threshold would suggest insufficient response effort (89). The means ranged from 1.79 (W3) to 1.90 (W4), with standard deviations between 0.75 and 0.83, reflecting adequate variability in responses. Skewness and kurtosis indices indicated that the item distributions approximated normality, although with a slight negative skew and leptokurtic tendency (90).

**Table 2:** Item analysis of the WHO-5 well-being index.

Ítem	Response Percentage				Mean	SD	Skewness	Kurtosis	CITC	If Item Deleted		h^2^	External Criterion	*p*-value
	0	1	2	3						Cronbach’s Alpha	McDonald’s ω			
W1	0.9%	34.8%	40.4%	23.9%	1.87	0.78	0.11	−1.10	0.64	0.81	0.81	0.58	0.21	< 0.001
W2	1.4%	33.7%	45.2%	19.7%	1.83	0.75	0.09	−0.83	0.66	0.80	0.80	0.63	0.27	< 0.001
W3	2.6%	38.7%	35.4%	23.3%	1.79	0.83	0.12	−1.03	0.70	0.79	0.79	0.63	0.26	< 0.001
W4	0.9%	31.0%	45.5%	22.5%	1.90	0.75	0.04	−0.93	0.68	0.79	0.80	0.57	0.28	< 0.001
W5	4.1%	31.5%	40.7%	23.8%	1.84	0.83	−0.12	−0.79	0.52	0.84	0.84	0.44	0.35	< 0.001

External criterion = Family functioning; h^2^ = Communality; CITC = Corrected Item-Total Correlation.

In terms of psychometric quality, the corrected item-total correlation (CITC), communality (h^2^), and the impact of deleting each item on the internal consistency of the instrument were evaluated. CITC values ranged from 0.52 (W5) to 0.70 (W3), indicating that most items adequately contribute to the overall construct (84, 91). Communalities were all above 0.30, indicating shared variance among items (92). The deletion of any item did not significantly affect internal consistency coefficients (Cronbach’s *α* and McDonald’s *ω*), which remained high across all cases, evidencing the reliability of the instrument (80, 93). Finally, correlations with the external criterion (Family functioning) were examined, showing significant associations (*p* < 0.001) for all items, supporting the criterion validity of the WHO-5.

### Validity evidence based on the internal structure of the WHO-5 in Peruvian older adults

Table 3 shows the results of the confirmatory factor analysis (CFA) of the WHO-5 well-being index, using the WLSMV estimator, which is appropriate for ordinal data. The global fit indices indicate an excellent fit of the proposed unidimensional model. The chi-square statistic divided by degrees of freedom (*χ*^2^/df = 3.73) is acceptable and consistent with reasonable fit. The Comparative Fit Index (CFI = 0.998) and Tucker–Lewis Index (TLI = 0.995) are above the 0.95 threshold, providing evidence of very good model fit. The error indices, both SRMR (0.03) and RMSEA (0.06), are also within the recommended values (< 0.08 and < 0.06, respectively), suggesting a low level of discrepancy between the model and the data (83, 94, 95).

**Table 3:** Confirmatory factor analysis (WLSMV Estimator) of the WHO-5 well-being index.

	X^2^/gl	CFI	TLI	SRMR	RMSEA
Model	3.73	0.998	0.995	0.03	0.06

	Instrument	Ítem	λ
Factor loadings of the WHO-5 well-being index items	WHO	W1	0.80
		W2	0.81
		W3	0.83
		W4	0.82
		W5	0.63

**Figure 1:**
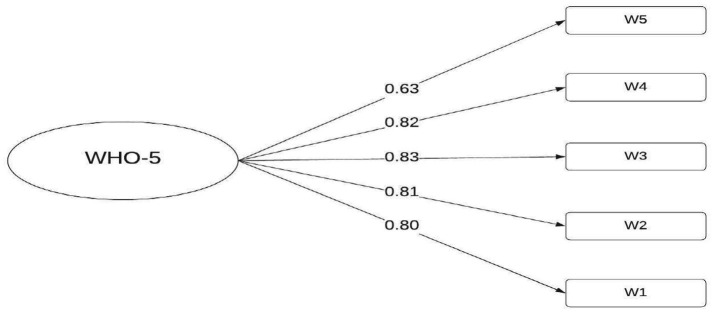
Path diagram of the CFA of the WHO-5 well-being index.

Regarding the factor loadings (*λ*), these reflect the contribution of each item to the overall construct of general well-being. All factor loadings are statistically significant and above the recommended threshold of 0.40, ranging from 0.63 (W5) to 0.83 (W3). This indicates that all items are adequate indicators of the latent construct, although item W5 shows a relatively lower loading, suggesting lower saturation compared to the other items. These results support the unidimensional structure of the WHO-5 and confirm the factorial validity of the instrument in the analyzed sample. This analysis provides strong evidence for its use in measuring general well-being (Figure 1).

### Factorial invariance of the WHO-5 by sex in Peruvian older adults

Table 4 presents the results of the factorial invariance analysis of the WHO-5 well-being index conducted by sex. This analysis evaluates whether the underlying factorial model is equivalent across groups, allowing valid comparisons of scores between them. Five levels of invariance were analyzed: configural, metric, scalar, partial scalar (strict), and structural. The configural model, which assesses equivalence in the factorial structure, showed good fit (CFI = 0.987; RMSEA = 0.123), indicating that the structure of the model is consistent across groups. In the metric model, where equality of factor loadings is assumed, a chi-square change was observed (Δ*χ*^2^ = 5.747, *p* < 0.001), with minimal changes in fit indices (ΔCFI = 0.001; ΔRMSEA = 0.018). This suggests that the relationships between items and the latent construct are similar across sexes.

**Table 4:** Fit indices of the factorial invariance analysis of the WHO-5 well-being index (original 5-item model) by sex.

Original 5-item model									
Según sexo	*χ*^2^	Δ*χ*^2^	gl	Δgl	p	CFI	ΔCFI	RMSEA	ΔRMSEA
Configural	59.58	…	10	…	…	0.987	…	0.123	…
Metric	65.32	5.75	14	4	***	0.986	0.001	0.105	0.018
Scalar	73.18	7.85	23	9	***	0.987	0.001	0.081	0.024
Partial scalar (estrict)	73.18	0.00	23	0	***	0.987	0.000	0.081	0.000
Structural	72.54	0.64	25	2	***	0.987	0.000	0.076	0.005

In the scalar model, which assumes equality of both factor loadings and intercepts, the changes in indices were also minimal (ΔCFI = 0.001; ΔRMSEA = 0.024), supporting the comparability of scores between sexes. The partial scalar (strict) model added equality in residual variances, without changes in fit (ΔCFI = 0.000; ΔRMSEA = 0.000). Finally, the structural model, which evaluates equality in the variance of the latent construct, maintained adequate fit (CFI = 0.987; RMSEA = 0.076; ΔCFI = 0.000). Taken together, these results support the factorial invariance of the WHO-5 by sex, allowing valid comparisons of general well-being scores across groups (84, 91, 96).

### Validity evidence of the WHO-5 in relation to other variables among Peruvian older adults

**Figure 2:**
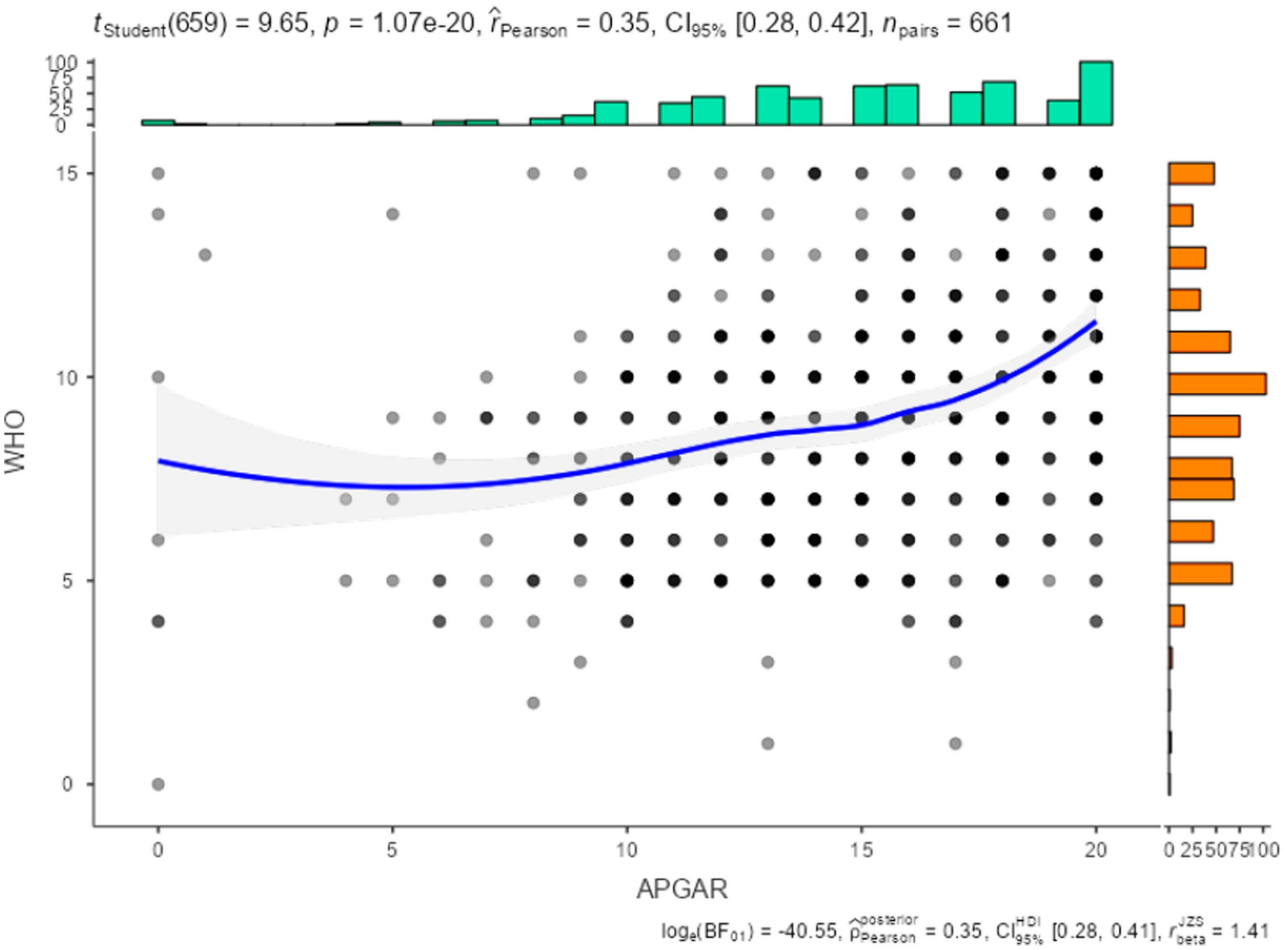
Correlation between WHO-5 and APGAR.

Table 5 and Figure 2 show the correlation between the Family Functioning scale (APGAR) and the WHO-5 well-being index, revealing a statistically significant and positive correlation (*r* = 0.35). This provides evidence of convergence between the constructs measured by the two instruments.

**Table 5:** Evidence of relationship with an external variable for the WHO-5 well-being index.

Instrument		Family functioning (APGAR)
General well-being (WHO-5)	Pearson’s r	0.35
	df	659
	p	< 0.001

**Figure 3:**
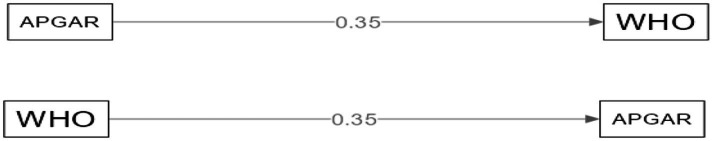
Path analysis evaluating the predictive capacity of both variables on each other. Funcionalidad familiar (APGAR); Bienestar general (WHO-5).

Figure 3 shows a path analysis conducted to evaluate the predictive capacity between two variables: family functioning (measured with the APGAR scale) and general well-being (measured with the WHO-5). In both directions (from APGAR to WHO-5 and from WHO-5 to APGAR), a path coefficient of 0.35 was observed, *p* < 0.001, *R*^2^ = 0.124 (12%). This indicates a moderate, positive, and bidirectional association between the two variables. These results suggest that family functioning influences general well-being and vice versa, to a similar extent. At the same time, this may indicate that the two variables are highly interrelated reciprocally, although it does not necessarily imply bidirectional causality.

### Reliability of the WHO-5 in Peruvian older adults

Table 6 presents the reliability indices of the WHO-5 well-being index. Cronbach’s alpha, with a value of 0.84, indicates a high level of internal consistency, reflecting strong interrelations among the items and a consistent measurement of the general well-being construct. Similarly, McDonald’s omega, also with a value of 0.84, demonstrates a high level of reliability, confirming the robustness of the instrument in measuring this construct. In all cases, the values exceed 0.70, which is considered acceptable (97–99).

**Table 6:** Reliability of the WHO-5 well-being index.

Scale	Cronbach’s Alpha	McDonald’s ω
WHO-5	0.84	0.84

The table presents the general percentiles corresponding to the scores of the WHO-5 well-being index. Percentiles allow for the interpretation of the score distribution in the sample, facilitating the relative comparison of individual values with the rest of the evaluated population. These data help situate individuals within the overall distribution of well-being in the population and are useful for classifying levels of well-being according to percentile ranges (Table 7).

**Table 7:** Percentiles of the WHO-5.

Percentil	WHO-5
1	3.6
10	5
20	6
30	7
40	8
50	9
60	10
70	11
80	12
90	14
99	15

Taken together, the results provide evidence that the WHO-5 demonstrates adequate levels of reliability and validity in the sample of Peruvian older adults. The factor analysis confirmed its unidimensional structure, factorial invariance ensured valid comparisons between sexes, and associations with family functioning supported its convergent validity. In addition, percentiles provide useful benchmarks for interpreting individual scores. These findings support the use of the WHO-5 as a brief and reliable instrument for assessing general well-being in this population.

## Discussion

The results of this study demonstrate that the WHO-5 exhibits solid psychometric properties among Peruvian older adults, confirming its suitability as a brief measure of subjective well-being. Item analysis showed adequate dispersion, absence of extreme biases, and high homogeneity indices, supporting the quality of the items and their internal coherence (46, 80, 84, 90, 93). These findings are consistent with research underscoring the usefulness of the WHO-5 for reliably capturing states of well-being in diverse populations (63, 64).

Confirmatory factor analysis revealed an excellent fit for the unidimensional model, in line with the theoretical framework of the instrument and with international validations that highlight its cross-cultural validity (38, 40, 43, 47, 48). Furthermore, its consistency with studies that have demonstrated clinical utility across various conditions (28, 49, 50) reinforces its value for monitoring mental health and well-being in older adults.

Convergent validity, reflected in the significant correlations between the WHO-5 and family functioning, confirms the influence of the immediate social environment on older adults’ well-being (100, 101). These results align with research emphasizing the role of social support, community, and family networks as key determinants of quality of life in old age (7, 9, 102, 115).

Factorial invariance across sex supports the equivalence of the instrument and indicates that well-being differences are not explained by gender, but rather by contextual factors such as social, economic, and community resources, in accordance with international literature (17, 21, 22, 62, 103, 104). This reinforces the relevance of using the WHO-5 in comparative studies and in the development of public health policies on mental health targeting older adults in the Peruvian context.

In sum, this study not only provides evidence of the validity and reliability of the WHO-5 in a sample of Peruvian older adults, but also contributes to filling a gap in the regional literature, given that most prior studies have focused on general populations or linguistic adaptations without a specific emphasis on aging.

### Theoretical and practical implications

The findings reinforce the conceptualization of subjective well-being as a unidimensional construct, shaped by family, social, and community factors. They also support the WHO framework on healthy aging (73), emphasizing that well-being is not solely the absence of disease but also involves social resources, resilience, and community participation (11, 12).

Practically, the WHO-5 proves to be a brief and effective tool for depression screening and well-being promotion in clinical, hospital, and community settings. It can guide preventive interventions, support early risk identification, and inform public health policies aimed at improving the quality of life in older adults.

### Limitations and future directions

This study presents limitations. First, a non-probabilistic sample from Metropolitan Lima restricts the generalizability of the results. Second, older adults with specific clinical diagnoses such as depression, Alzheimer’s, Parkinson’s, or other neurodegenerative conditions were not included, limiting evidence on discriminant validity. Third, no clinical external criterion was applied, constraining conclusions about diagnostic accuracy.

Future studies should validate the WHO-5 in clinical and multi-regional populations, assess sensitivity with ROC curves, and explore longitudinal stability. Cross-cultural comparative research could further clarify contextual and cultural differences in the perception of well-being (7–9).

Finally, a content-focused approach and coherence of the construct under evaluation are recommended through qualitative strategies such as cognitive interviews, in-depth interviews, and focus groups. These techniques allow for the identification of ambiguities, the interpretation of how participants understand the items, and the provision of validity evidence based on content (105, 106). Furthermore, in accordance with the *Standards for Educational and Psychological Testing* (77), the incorporation of qualitative data supports the collection of validity evidence based on response processes by clarifying the correspondence between the theoretical intent of the item and the respondent’s interpretation. This approach not only strengthens the quality of the instrument but also ensures its cultural and contextual relevance across diverse populations, thereby optimizing the validity of inferences derived from its use.

## Conclusion

In summary, the WHO-5 well-being index demonstrated strong validity, reliability, and measurement invariance in a sample of Peruvian older adults. The instrument confirmed its unidimensional structure, with adequate internal consistency and convergent validity with family functioning. These findings support its use as a culturally appropriate, brief, and reliable tool for assessing general well-being in older adults.

## Data Availability

The raw data supporting the conclusions of this article will be made available by the authors, without undue reservation.
